# Mercury in the Surface Soil and Cassava, *Manihot esculenta* (Flesh, Leaves and Peel) Near Goldmines at Bogoso and Prestea, Ghana

**DOI:** 10.1007/s00128-012-0849-7

**Published:** 2012-10-07

**Authors:** A. Adjorlolo-Gasokpoh, A. A. Golow, J. Kambo-Dorsa

**Affiliations:** 1 Science Department, School of Applied Science, Takoradi Polytechnic, Takoradi, Ghana; 2 Department of Chemistry, School of Nuclear and Allied Sciences, University of Ghana, Legon, Ghana; 3 Department of Chemistry, University of Cape Coast, Cape Coast, Ghana

**Keywords:** Total mercury, Native gem, Cassava, Amalgam

## Abstract

Mercury amalgamation is used indiscriminately in the recovery of gold by small-scale native gem winners in Ghana. Mercury is released into the environment in the form of wastewater, tailing and vapor from the roasting of amalgam to separate gold. The study looked at the levels of total mercury concentration in surface soil and cassava crop from farms located within the vicinities of Bogoso and Prestea Goldmines. The surface soil total mercury concentrations ranged between 125.29 and 352.52 μg/kg whiles cassava had between 66.60 and 195.47 μg/kg. The results showed proportionately more deposits at higher distances in 15−30 cm soil zone and less deposits at higher distances on leaves with relatively high uptake of the metal occurred at higher distances from the mines into the peels. These results suggest serious mercury pollution to the surface soil and the cassava crop but the speciation exercise showed that mercury is not in the free state, rather bound to hydroxides and organic compounds as complexes.

Ghana has been endowed with diverse mineral resources such as gold, diamond, bauxite and manganese dioxide. Mining had been the single industry attracting overseas investors to Ghana. Modern gold mining has taken mechanical- and chemical-intensive dimension, in which hundreds of tons of rock and gold are moved and processed for every ounce of gold. At Bogoso and Prestea, gold is mined from a series of open pits and from limited underground operation on the concession by large-scale mining companies. Small-scale native gem winners popularly called ‘galamsey’, widely spread all over the area, and mine from the open pits and tailing dams. These small-scale native gem winners mainly use mercury in the extraction of gold by amalgamation.

Mercury has high vapour pressure and vapourises easily into the atmosphere. It may condense on particulate matter in the atmosphere. The condensed mercury may fall back to the earth in precipitation, (rain, dew and fog) to contaminate soils, vegetation, humans and livestock. Mercury is a toxic heavy metal and of significant concern as an environmental pollutant (Sutton et al. 2002; Falandysz et al. 2012; WHO 1991). The extreme and pronounced toxicity of mercury and its environmental contamination have resulted in many episodes of human poisonings (Hisashi 2009; George 2001; Ibrahim et al. 2006). Mercury contaminates soil and waters (both surface and underground). Plants also absorb this toxic metal posing serious health problems to man since they are source of food to man. The uptake of the metal from both the atmosphere through leaf surfaces and from the soil through the roots may account for the elevated levels of the metal in the food crops grown in mining areas (Alloway and Ayres 1994; Essumang et al. 2007; Falandysz et al. 2002). Also, since the small-scale native gem winners (galamsey) are scattered over in the vicinities of the goldmines, it will be of importance to determine the mercury distribution in the vicinities of Bogoso and Prestea goldmines.

## Materials and Methods

The samples were collected from farms located within the vicinities of Bogoso and Prestea Gold mines. At each sampling site, three sub sites were located. Soil samples were collected by removing the top litter first, and a Teflon-coated soil auger was used to collect the samples into an already well-washed polyethylene container and sealed. The samples were collected randomly at depths corresponding to 0−5, 5−15 and 15−30 cm to cover the plough zone. The auger was washed with distilled water after sampling at each site to avoid cross contamination. Identification codes were written on each polyethylene container and conveyed to the laboratory. Nine surface soil samples were collected from each site. The corresponding depths of soil were thoroughly mixed on a clean piece of polyethylene sheet. The bulk was reduced by removing pieces of stones, leaves and roots, so that composite sample was retained.

Cassava tubers were uprooted, by first scrapping off the topsoil with a cutlass, from three plants at each site. A stainless steel knife was used to cut the tubers from their stems and adherent soil was removed gently. The cassava leaves were clipped with fingers from the same plants at more than one meter above the ground. All the cassava samples (tubers and leaves) were collected into separate polyethylene bags and sealed. Identification codes were written on all the bags as well as on the sealed stickers. All were conveyed to the laboratory.

In the laboratory, soil samples were spread out on a polyethylene sheets and freed of pieces of roots, dead leaves, pebbles and other foreign objects. Large lumps of soil were broken by hand. The samples were air-dried at room temperature to a constant weight. The dried samples were ground and homogenized in a porcelain mortar, sieved with 120 μm size mesh and made into composite sample. They were finally transferred into polyethylene containers sealed, labeled and stored at room temperature.

For cassava samples, fresh cassava tubers were individually washed gently and rinsed with distilled water, and peeled. The fresh peels were removed with a stainless knife and separated into the peridem (outer skin) and cortex with fingers. Each was chopped up separately into smaller pieces and dried to constant weight at 35°C temperature.

Fresh leaves were also chopped up into smaller pieces for easy drying. The dried leaves, flesh and peels of the cassava were oven-dried at 35°C to a constant weight. The samples were then ground and homogenized in a porcelain mortar and then sieved with 120 μm size mesh. All samples after sieving were transferred separated into polyethylene containers, labeled and stored at room temperature. The powdered peridem (outer skin) code named Peel-A and cortex code named Peel-B and flesh or core were separately analyzed. For mercury digestion, exactly 1.0 g of the treated soil sample was weighed into decontaminated 100 mL beakers. 5 mL double distilled water and 5 mL aqua regia were added respectively to the treated sample in the beakers. Each of them was mixed thoroughly and placed in a water bath for 2 min at 90°C. The beakers were removed and allowed to cool to room temperature. 50 mL double distilled water was added followed by 15 mL of 5 % w/v of BDH potassium permanganate solution to each sample. They were mixed thoroughly again and then placed in a water bath for 30 min at 95°C for complete oxidation of mercury in the soil samples. The samples were removed, allowed to cool to room temperature and 6 mL of 12 % w/v of BDH hydroxylamine hydrochloride solution were added to reduce the excess permanganate colour in the mixtures. The resulting solutions were filtered through pre-washed Whatman No. 1 filter paper into 100 mL graduated flasks, diluted to the marks with double distilled water, and then stored in the refrigerator to await analysis.

Using the cold vapour technique of the AAS, a blank containing 10 mL of concentrated 69 %−70.5 % AnalaR HNO_3_ in 100 mL of double distilled water, was first used to zero the AAS Shimadzu 6400AA pc equipped with mercury hollow cathode lamp. The digest, a carrier solution of 5 % v/v of 98 % AnalaR H_2_SO_4_ and reducing agent 1.1 % w/v BDH SnCl_2_·2H_2_O in 3 % v/v of 36 % AnalaR HCl were mixed in a mixing chamber. This was done by bubbling nitrogen gas from a pump, resulted in the generation of the free mercury from the digest in the presence of reducing agent in the mercury vapour generator. The mercury vapour generated then swept into the absorption cell mounted on the AAS (Shimadzu 6400AA pc) and measurements made automatically. The cell was aligned in the path of mercury hollow cathode lamp operating at 4 mA and monitored at the 253.7 nm resonance lines. During the analysis, blank solution was analyzed intermittently to zero the instrument after every 20 samples. Analyses were done in triplicates by sucking 5 μL volume of the solution containing the metal in the mixing chamber. Aerating the elemental mercury into the path of the mercury lamp, the mercury atoms absorb the wavelength and the absorption measured by the instrument is proportional to the mercury atoms in the path; hence the mercury concentration of the prepared solution in μg/L. Calibration curve for mercury was prepared from mercury standard solutions and samples read from this curve.

One gram of accurately weighed cassava tissue samples were put into 100 mL decontaminated beakers, 4 mL of concentrated 98 % AnalaR H_2_SO_4_ followed by 1.0 mL of 69 %−70.5 % AnalaR HNO_3_ were added to each sample and then placed in water bath at 80°C for 30 min for samples to dissolve completely. The samples were then cooled to 4°C in an ice bath and 15 mL of 5 % w/v BDH potassium permanganate solution were added followed by 8 mL of 5 % w/v BDH potassium persulphate solution to oxidize all alkyl mercury and other organic-mercury in solution to 2+state. The samples were again returned to the water bath and digested for an additional 30 min at 90°C. The samples were removed from water bath, allowed to cool to room temperature and 6 mL of 12 % w/v of BDH hydroxylamine hydrochloride solution were added to reduce the excess permanganate colour in solution. The resulting solutions were the filtered through pre-washed Whatman No. 1 filter paper into 50 mL volumetric flasks and diluted to the mark using double distilled water and were stored in refrigerator to await analysis. AAS analyses were done in the same way as soil samples described above.

The reproducibility tests carried out in replicates gave a mean of 1.99 μg/L, standard deviation of 0.021. The coefficient of variation and standard error were 1.042 % and 0.007 for 2.00 μgHg per litre of added mercury standard. The recovery test for spiked standards ranged between 99.3 % and 102.0 % for amounts between 0.5 and 2.5 μg/L.

## Results and Discussion

Mercury levels in the 0−5 cm soil zones of the area except Prestea Old Mine values were lower than those of the 5−15 and 15−30 cm soil zones (Table 1). But most of 15−30 cm soil zone mercury levels were higher than 0−5 and 5−15 cm soil zones. These observations indicate that if the source of mercury were aerial then precipitation might have leached the mercury to the deeper soil zones. It might partly be due to porosity of the topsoil that enhanced leaching of the metal and the metal’s inability to adsorb onto surface soil. At Prestea, the 0−5 and 5−15 cm soil zones had higher mercury than the 15−30 cm a proof that source was aerial.

**Table 1:** Mercury levels in surface soil near Bogoso/Prestea gold mine

Sampling site	Distance (km)	Depth (cm)	Concentration of mercury (μg/kg)				Mean	SD
			1	2	3	4		
Entrance of Bogoso Gold Ltd.	1.5	0−5	256.56	120.51	251.49	243.26	217.96	19.36
		5−15	166.10	156.41	271.28	231.73	206.41	54.67
		15−30	261.24	213.18	283.26	262.43	255.03	29.67
Dumasi Site A	5.5	0−5	197.91	162.05	206.53	354.67	230.29	85.13
		5−15	156.43	428.79	228.39	369.26	295.72	111.22
		15−30	131.44	301.03	156.57	243.10	208.04	87.02
Dumasi Site B	6.0	0−5	−	165.72	102.97	184.64	151.11	42.75
		5−15	−	146.95	345.35	223.43	238.58	100.06
		15−30	−	150.71	351.09	263.68	255.16	100.46
Bogo River Bank	8.4	0−5	93.63	129.01	121.80	156.71	125.29	25.92
		5−15	292.50	149.73	197.24	198.88	209.59	59.79
		15−30	348.17	178.92	251.49	189.46	242.01	77.68
Prestea Ankobra Before Bridge	14.0	0−5	300.55	231.33	399.42	334.76	316.52	70.04
		5−15	276.21	399.23	294.29	298.31	317.01	55.65
		15−30	206.29	165.24	227.27	256.97	213.94	38.56
Prestea Old Mine	16.0	0−5	392.46	320.34	330.56	366.72	352.52	33.24
		5−15	325.91	301.12	297.21	310.08	308.58	12.75
		15−30	256.63	283.80	223.33	284.72	262.12	28.96

The general trend observed for mercury levels in the cassava tissues (Table 2), the leaves registered more of the mercury concentrations than the peels and flesh in Bogoso area. This suggests the source of the mercury might be aerial. The mercury levels decreased with distance except at Prestea sites, for some tissues but there was some discrepancies with the trend. This might probably be due to high mercury levels obtained in the soil from Prestea sites.

**Table 2:** Mercury levels in cassava tissues near Bogoso/Prestea gold mine

Sampling site	Distance (km)	Sample type	Concentration of mercury (μg/kg)				Mean	SD
			1	2	3	4		
Entrance Of Bogoso Gold Ltd.	1.5	Leaves	176.99	146.52	127.18	145.62	149.08	20.63
		Flesh	150.28	159.81	98.51	101.19	127.45	32.12
		Peel-A	77.91	68.28	43.29	76.92	66.60	16.13
		Peel-B	82.72	85.43	76.17	58.14	75.62	12.28
Dumasi Site A	5.5	Leaves	153.46	125.37	147.72	127.89	138.61	14.07
		Flesh	184.78	154.61	165.97	165.31	167.67	12.54
		Peel-A	98.62	96.73	89.67	72.12	89.29	12.07
		Peel-B	101.77	102.86	97.28	91.10	98.25	5.35
River Bogo River Bank	8.4	Leaves	126.71	211.79	112.33	92.37	135.80	52.58
		Flesh	109.82	168.18	83.54	126.78	122.08	35.51
		Peel-A	110.97	92.82	125.14	92.89	105.46	15.66
		Peel-B	96.76	56.99	99.13	62.13	78.75	22.28
Prestea Ankobra Before Bridge	14.0	Leaves	134.72	108.14	89.72	120.93	113.38	19.15
		Flesh	175.41	169.64	126.41	136.99	152.11	24..08
		Peel-A	109.26	124.21	76.30	176.54	121.58	41.75
		Peel-B	162.19	195.82	267.84	156.02	195.47	51.32

**Fig. 1:**
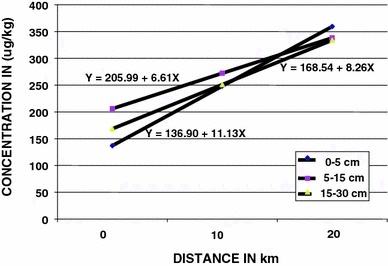
Linear regression of mean mercury levels in surface soil and distance from the mine

Statistically, mercury levels in surface soil from Bogoso Gold mine vicinity showed fairly strong positive linear correlations with distance from the mine and there was proportionately less leaching of the metal towards higher depth (Fig. 1). The mercury levels were higher than the normal background level of 0.5−50 ppb (McBride 1994). There is therefore an indication of mercury pollution in the vicinity of Bogoso mine.

**Fig. 2:**
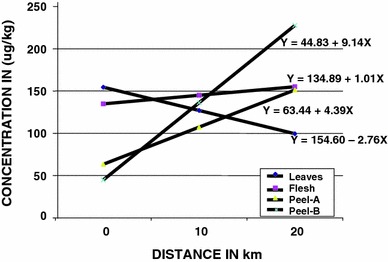
Linear regression of mean mercury levels in cassava distance from Bogoso goldmine

Mercury levels in cassava tissues from Bogoso mine vicinity showed strong linear correlations with distance from the mine, except flesh (pith) showed a weak linear correlation with distance (Table 3, Fig. 2). The negative linear correlation of levels with distance for cassava leaves confirmed that precipitation in the area might have washed the metal deposits off the soil and consequently into the 15−30 cm soil zones of the area. There was proportionately less deposit of the metal at high distances on the leaves whilst relatively high uptake of the metal occurred at higher distances from the mine, into the peels.

**Table 3:** Linear regression of distances from Bogoso gold mine and mercury levels in the surface soil and cassava crop

Sample	Linear regression equation	Correlation coefficient
Soil		
0–5 cm	Y = 136.90 + 11.13X	0.69
5–15 cm	Y = 205.99 + 66.12X	0.72
15–30 cm	Y = 168.54 + 8.27X	0.40
Cassava		
Leaves	Y = 154.60–2.76X	-0.98
Flesh	Y = 134.89 + 1.01X	0.25
Peel-A	Y = 63.44 + 4.39X	0.98
Peel-B	Y = 44.83 + 9.14X	0.85

*Y* concentration of mercury, *X* distance from the mine

The mercury levels in both surface soil and cassava tissues were in the range of 66.60−352.52 μg/kg. This might be introduced into the vicinity by the indigenous gold mining activities around the mine’s tailing dams and its concessions. The regression analysis on the surface soil mercury levels showed proportionately more deposits at higher distances in 15−30 cm soil zones, while cassava tissues showed proportionately less deposits on the leaves. The surface soil however, is polluted in the area, root crops in the vicinity could be poisoned since cassava leaves contain higher mercury, its use for stew may cause accumulation which may reach or exceed toxic limits. The levels in mercury measured in the soil and cassava were higher than those measured in Dunkwa-On-Offin by Golow and Adzei (2002), this might be due to more ‘galamsey’ activity in Bogoso area.
